# An insight into gut microbiota and metabolites in the mice with adenomyosis

**DOI:** 10.3389/fcimb.2023.1075387

**Published:** 2023-02-27

**Authors:** Peipei Chen, Kun Wang, Mingyan Zhuang, Xianyun Fu, Shidan Liu, Minmin Chen, Ya Lei

**Affiliations:** ^1^ Third-Grade Pharmacological Laboratory on Chinese Medicine Approved by State Administration of Traditional Chinese Medicine, Medical College of China Three Gorges University, Yichang, Hubei, China; ^2^ Obstetrics and Gynecology Department of Maternity and Child Health Care Hospital, Three Gorges University, Yichang, Hubei, China

**Keywords:** adenomyosis, gut microbiota, fecal metabolites, sex hormone, glutathione

## Abstract

**Background:**

Adenomyosis (AM) is a benign uterine disease characterized pathologically by the invasion of endometrial tissue into the myometrium. The pathogenesis of AM is still far from clear. Although the gut microbiome and metabolomics are thought to contribute to a variety of diseases, the role of them in AM has not been revealed.

**Objective:**

To investigate changes in the gut microbiota and derived metabolites in AM mice.

**Method:**

Female ICR mice were randomly assigned to AM and control groups, and pituitary transplantation was employed to perform AM modeling. Then, the fecal samples were obtained for microbial (16S rRNA gene sequencing) and metabolomic (liquid chromatography mass spectrometry, LC-MS) analysis.

**Result:**

The results of gut microbiota analysis showed that the intestinal microbiota composition of AM mice was altered. The ratio of *Firmicutes/Bacteroidetes* and the relative abundance of *Lactobacillus* in AM group increased compared with the control group. Sixty differential expressed metabolites were identified in intestinal metabolites, mainly involved in steroid hormone biosynthesis, cysteine and methionine metabolism, and alanine, aspartate, and glutamate metabolism. Further, correlation analysis verified that *L*-methionine and *L*-cystine were negatively correlated with *Bacteroides* and positively correlated with *Desulfovibrio*. The Pregnenolone, Androsterone glucuronide, and Testosterone glucuronide were negatively correlated with *Unidentified_Ruminococcaceae* and *Alistipes*, whereas they positively correlated with *Bacteroides*.

**Conclusion:**

AM mice have a unique gut microbiome and intestinal metabolites.

## 1 Introduction

Adenomyosis (AM), pathologically characterized by the migration of the myometrium into endometrial tissue, is a significant threat to the woman’s health due to its high incidence (Bourdon et al., 2021). The typical manifestations of AM, including abnormal uterine bleeding, pelvic pain, dysmenorrhea, and infertility, seriously reduce the quality of women’s life and work (Stratopoulou et al., 2021; Moawad et al., 2022). Although the pathogenesis of AM remains controversial, sex steroid hormone aberrations, including estrogen and progesterone, as well as immune disorders, are widely recognized to be responsible for the increased cell proliferation in AM (Vannuccini et al., 2017).

The gut microbiome is the collection of all the microorganisms in the human gastrointestinal tract (Ni et al., 2020). Recent evidence suggested that gut microbes act as an extra organ by actively participating in shaping and maintaining human physiology (Motiani et al., 2020). Alterations in gut microbial composition and function can regulate gut permeability, digestive metabolism, and immune responses. AM is an inflammatory-associated and estrogen-dependent disease (Barcena et al., 2013). The altered balance of gut microbes was confirmed, resulting in a pro-inflammatory state (Gomaa, 2020). Besides, studies have shown that the gut microbiota can also affect estrogen levels (Motiani et al., 2020; Song et al., 2020; Qi et al., 2021). Although differential microbiota has been identified in endometriosis (Ata et al., 2019; Huang et al., 2021; Svensson et al., 2021), the relationship between gut microbes and AM has not been revealed.

As a non-neoplastic disease, the metabolites with altered levels are particularly involved in immune activation, cell proliferation, and cell migration in AM (Bourdon et al., 2021). Therefore, information-riched metabolic profiles are increasingly attracting the attention of researchers. With the development of high sensitivity and specificity of mass spectrometry techniques, exploring the potential mechanisms by metabolomic analysis becomes feasible (Yang et al., 2017a). It has been found that serum differential metabolites contribute to immune activation in AM patients, resulting in the upregulation of cell proliferation and migration (Bourdon et al., 2021). Other researchers confirmed that the differential metabolites in the myometrium of AM are associated with AM inflammatory response, oxidative stress, cell proliferation, apoptosis, and energy metabolism processes (Song et al., 2022). The gut microbiota, involved in a variety of metabolic processes including glucose, amino acid, bile acid, and choline, may be responsible partly for the formation of the pathogenic microenvironment in AM.

In this study, the altered intestinal microbiota composition of AM mice was demonstrated by 16s rRNA sequencing, and sixty differential expressed metabolites were also identified in intestinal metabolites. Besides, exploring the possible correlation between the differential intestinal metabolites and alteration of the gut microbiota further reveal their underlying mechanisms in the progress of AM.

## 2 Materials and methods

### 2.1 Experimental animals

ICR mice (7 weeks old) were obtained from Beijing Weitong Lihua Laboratory Animal Technology Co., Ltd (NO. 2016-0006). Animal welfare and experimental procedures conformed to the Guidelines for the Care and Use of Laboratory Animals of China Three Gorges University. All animals had a normal diet and circadian rhythm during the experiment. The experimental protocol was approved by the Ethical Committee in Research Medical College of China Three Gorges University of Medical Sciences (NO. 20190801).

### 2.2 Construction of the AM mouse model

16 female mice aged seven weeks were randomly assigned to the control group (n = 8) and the AM group (n = 8). In this experiment, the pituitary transplantation method was used for the modeling of AM (Marquardt et al., 2020). The female mice were injected intraperitoneally with propofol for anesthesia (Xian Nippon Pharmaceutical Co. Ltd. Xian, China, No. H19990282, 100 mg•kg^-1^). Then, a 2 cm longitudinal incision was made to the right of the lower abdomen. The pituitary acquired from the age matched male mice were injected into the right uterus of mice by trocar. Before closing the incision, gentamicin solution (0.25 ml, 20000 units/ 20 g) was dropped into the enterocoelia.

### 2.3 Sample collection

After six weeks, all the mice were sacrificed by cervical dislocation. The right uterine of the mice were obtained, and feces from the colons were collected. Both uterine and fecal samples were stored at -80 °C.

### 2.4 HE staining

After fixed by 4% paraformaldehyde, the uterine tissues were embedded in paraffin and sectioned at 5 μm. Histopathological alterations were observed by hematoxylin and eosin (HE) staining.

### 2.5 16s rRNA sequencing and data processing

The CTAB/SDS method was used to extract total genome DNA from fecal samples. The V3-V4 region of the bacteria 16S rRNA gene was targeted and PCR amplified with primer 341F (5′-CCTAYGGGRBGCASCAG-3′) and 806R (5′-GGACTACNNGGGTATCTAAT-3′). All PCR reactions were carried out with Phusion^®^ High-Fidelity PCR Master Mix (New England Biolabs). GeneJETTM Gel Extraction Kit (Thermo Scientific) was used to purify mixture of PCR products. Then, they were analyzed by Illumina NovaSeq6000 for machine sequencing.

Raw tags were merged by the reads of each sample with FLASH (V1.2.7) software. The raw tags were quality filtered using the QIIME (V1.9.1) quality controlled procedure to produce the clean tags. Uparse software (V7.0.100) was used for sequences analysis. Sequences with ≥97% similarity were clustered to the same OTUs. The OTUs sequences were classified by species annotation according to the silva SSUrRNA database (Quast et al., 2013). Alpha and beta diversities were calculated by QIIME (V1.9.1). Linear discriminant analysis (LDA) coupled with effect size (LEfSe) was applied to evaluate the differentially abundant taxon.

### 2.6 Fecal metabolome analysis

Fecal samples (100 mg) were ground with liquid nitrogen in the Eppendorf tubes, and added with 80% methanol and 0.1% formic acid. The mixtures were resuspended by well vortex, kept on the ice for 5 min, and subsequently centrifuged at 15000 g, 4 °C for 10 min. The supernatant (300 μL) was diluted with 150 μL of LC-MS grade water to 53% methanol conten and then was centrifuged at 15000 g, 4°C for 10 min. Finally, the supernatant was collected and injected into the LC-MS system for metabolomics analysis. The quality control (QC) samples mixed with all test samples in this study were used to evaluate the stability of the analytical system during operation to ensure the reliability of the results.

A Vanquish UHPLC system (Thermo Fisher, Germany) and a Qrbitrap Q Exactive™ HF mass spectrometer (Thermo Fisher, Germany) were used to perform LC-MS analyses. The data matrix, obtained by LC-MS analyses, was imported into a SIMCA version 14.0, Umetrics, Umea, Sweden). Firstly, principal component analysis (PCA) was performed to visualize the distribution of all samples. Then, orthogonal partial least squares discriminant analysis (OPLS-DA) was used to discriminate the metabolites of the two groups. By variable importance plot (VIP>1.0) and *P*-values (*P*<0.05), the differential metabolites responsible for discriminating between the two groups were identified. MetaboAnalyst 5.0 (https://www.metaboanalyst.ca/) was conducted to perform pathway analysis, and Impact > 0.1 was utilized as a screening threshold to identify probable metabolic pathways (Song et al., 2022).

### 2.7 Statistical analysis

All data were expressed as mean ± standard deviation. GraphPad Prism 8.0.1 was used to draw a comparison chart of gut microbial relative abundance at different levels, and unpaired t-test (SPSS 17.0) was used for comparison between groups. *P* < 0.05 was considered statistically significant. Spearman correlation coefficients between differential metabolites and bacterial genera were calculated using R language (Pheatmap package), and the data matrix was displayed as a heatmap.

## 3 Results

### 3.1 HE staining

**Figure 1:**
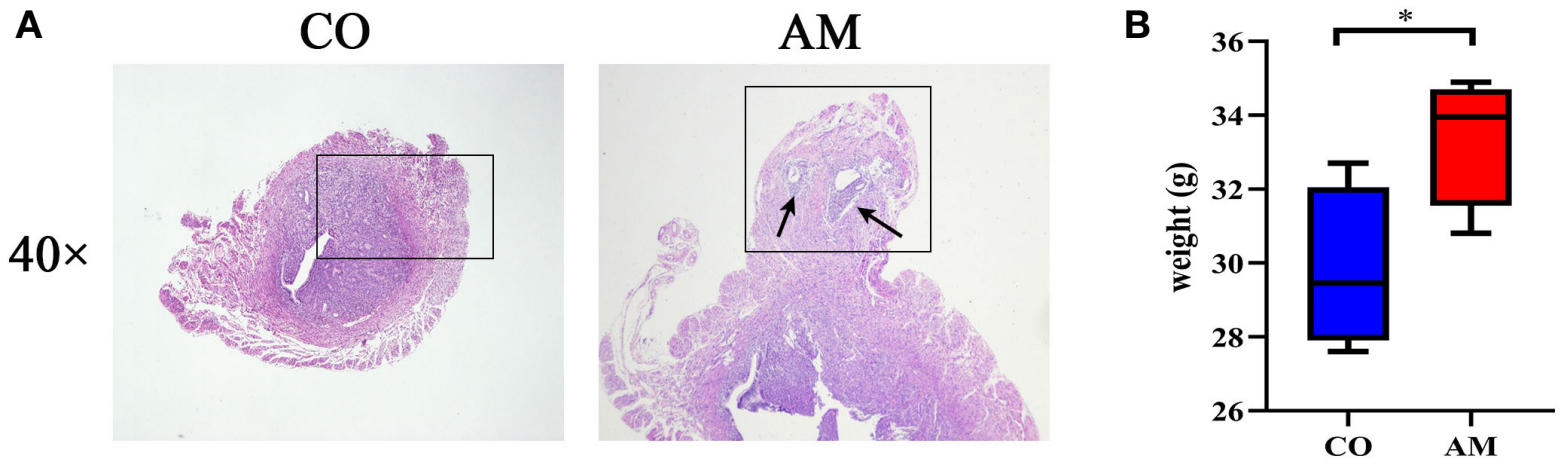
HE staining and body weight changes of mice in control and AM groups. **(A)** HE staining of transverse uterine sections in control group (left) and AM group (right). **(B)** Changes in body weight of mice after modeling (n=8 per group). CO, control group, AM, AM group. *P<0.05.

According to the results of the HE staining, the boundary between the endometrium and the myometrium in the control group was distinct. On the contrary, in the AM group, the glands and stromal cells of the endometrial layer invaded into the myometrium (black arrow), as shown in **Figure 1A**. The result indicated that the modeling of AM was successful.

### 3.2 Body weight changes in mice

The body weight of the mice changed after feeding for 6 weeks. However, significant weight gain occurred in the mice from the AM group in contrast with the control group (*P* = 0.044) (**Figure 1B**).

### 3.3 Composition of the gut microbiota in the mice with AM

**Figure 2:**
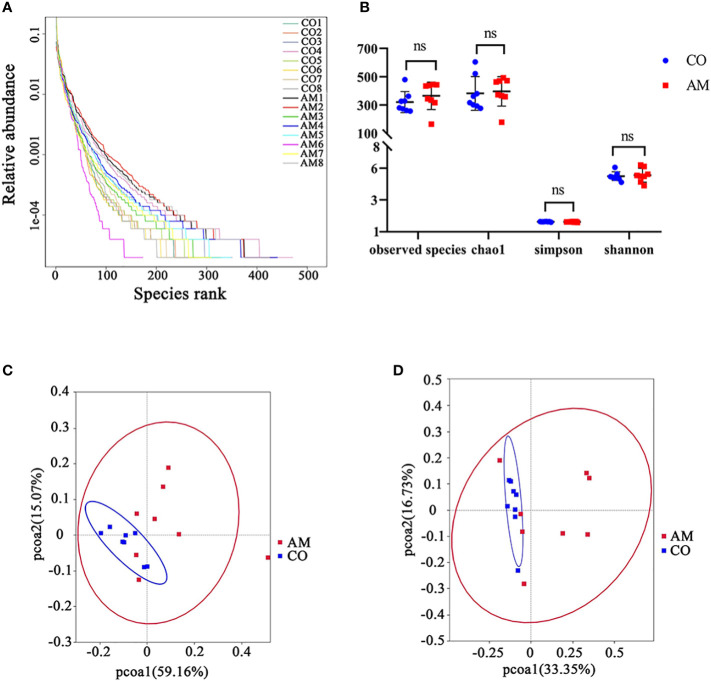
Diversity analysis of gut microbiota in control and AM groups. **(A)** Rank-abundance curves. The abscissa position of the extension endpoint of the sample curve was the number of OTU. **(B)** Boxplot of alpha diversity (Observed species, Chao1, Shannon, and Simpson index) for gut microbiota. Weighted uniFrac PCoA plots **(C)** and unweighted uniFrac PCoA plots **(D)**. PC1 and PC2 represented the two suspected influencing factors of microbial composition migration. The percentage represented the contribution of principal coordinate components to sample composition differences. The closer the two sample points were, the more similar the species composition of the two samples was. ns, not significantly different.

The results of 16S rRNA sequencing technology showed that the structure of the gut microbiota of AM mice has varied. After taxonomic assignment, 988 OTUs were obtained (**Supplementary Table S1**). The rank-abundance plot (**Figure 2A**) indicated that the gut microbiota of AM mice has changed. The results of Alpha diversity indiced that there was no significant difference between the two groups in Observed species, Chao1, Simpson, and Shannon. (Observed species index, *P*=0.32; Chao1 index, *P*=0.68; Simpson index, *P*=0.38; Shannon index, *P*=0.74; **Figure 2B**). The beta diversity of gut microbiota, based on the weighted and unweighted PCoA, revealed a separation of the control and AM groups (**Figures 2C**, **D**).

**Figure 3:**
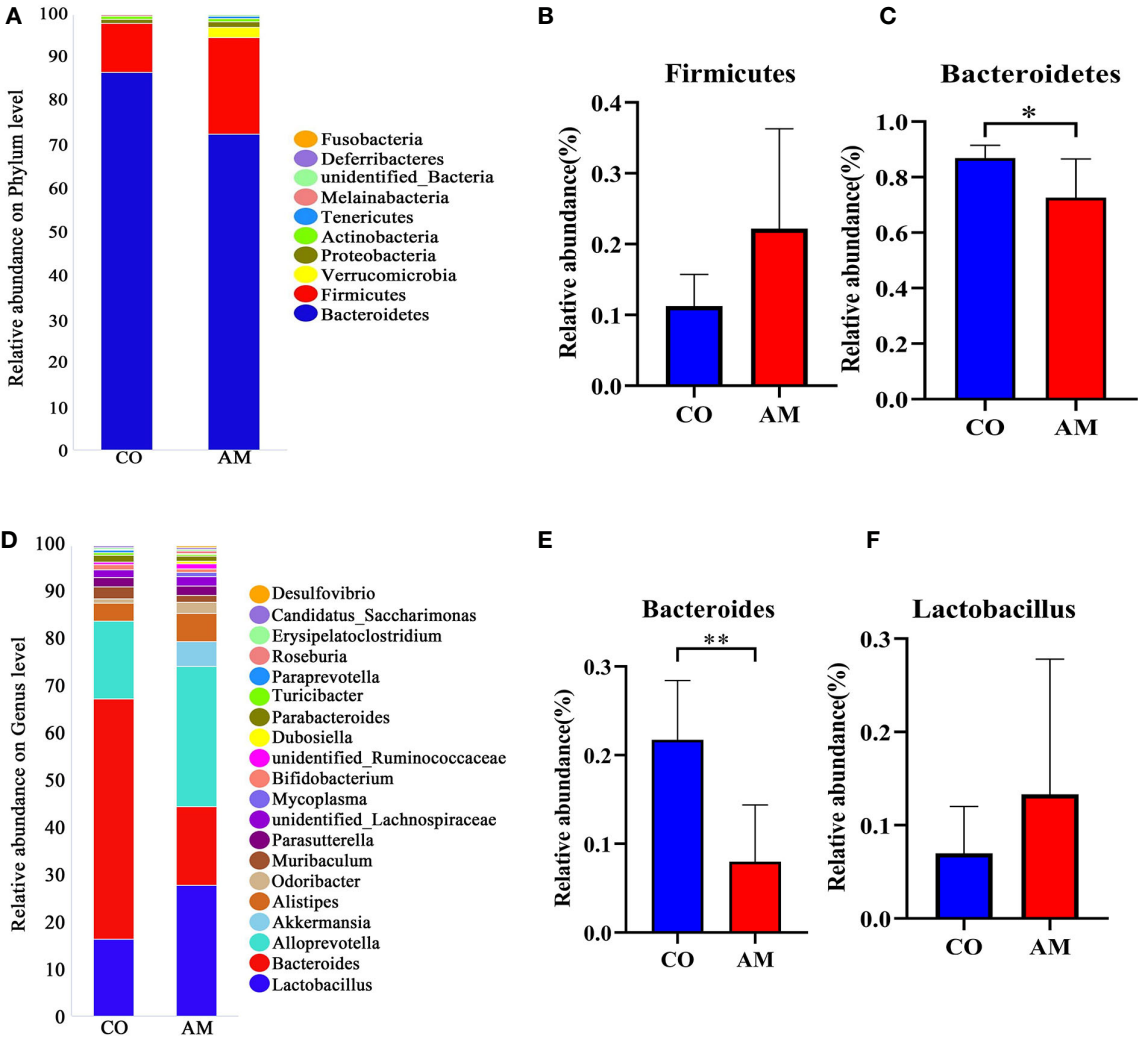
Composition of gut microbiota in control and AM groups. **(A)** Composition of species at phylum level. **(B)** The relative abundance of *Firmicutes* (n=8 per group). **(C)** The relative abundance of *Bacteroidetes* (n=8 per group). **(D)** Composition of species at genus level. **(E)** The relative abundance of *Bacteroides* (n=8 per group). **(F)** The relative abundance of *Lactobacillus* (n=8 per group). *, *P*<0.05, **, *P*<0.01.

At the phylum level (**Figure 3A**), the ratio of *Firmicutes*/*Bacteroidetes* in the AM group was increased compared to that in the control group (0.36 vs. 0.13, *P*=0.109). *Firmicutes* (*P*=0.055, **Figure 3B**) was enriched in the AM group compared to the control group, whereas *Bacteroidetes* (*P*=0.015, **Figure 3C**) was significantly more abundant in the control group. Among the top 20 most abundant genera (**Figure 3D**), *Bacteroides* (*P*=0.001, **Figure 3E**) was found to be significantly more abundant in the control group, while *Lactobacillus* exhibited increased relative abundance in the AM group (*P*=0.263, **Figure 3F**).

### 3.4 Characteristic bacterial analysis of gut microbiota in the mice with adenomyosis

**Figure 4:**
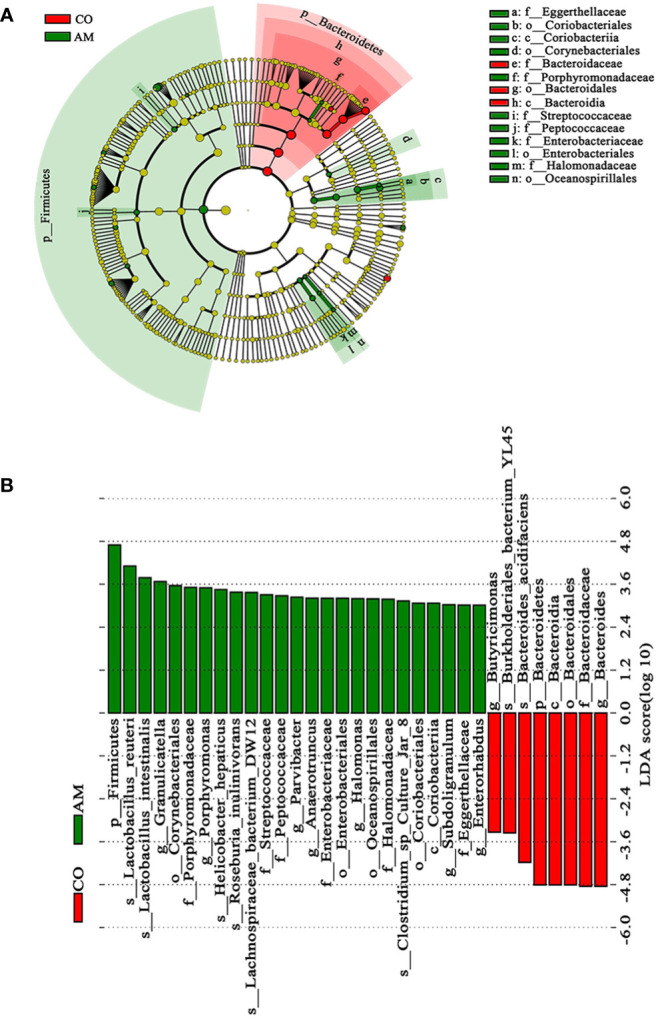
Linear discriminant analysis (LDA) integrated with effect size (LEfSe). **(A)** Cladogram indicating the phylogenetic distribution of microbiota. **(B)** The differences in relative abundance between the control and AM groups.

To distinguish the crucially different flora in the two groups, we performed LEfSe analysis. At every level of classification, from phylum to genus, significant species that greatly influenced the differences between the two groups were discovered by LDA score (**Figure 4A**). At the genus level, we found seven kinds of significantly characteristic bacteria (LDA>3.0), including *Granulicatella*, *Porphyromonas*, *Parvibacter*, *Anaerotruncus*, *Halomonas*, *Subdoligranulum*, and *Enterorhabdus*, which were more abundant in the AM group, compared with the control group (**Figure 4B**).

### 3.5 Fecal metabolomic characteristics of AMs mice

**Figure 5:**
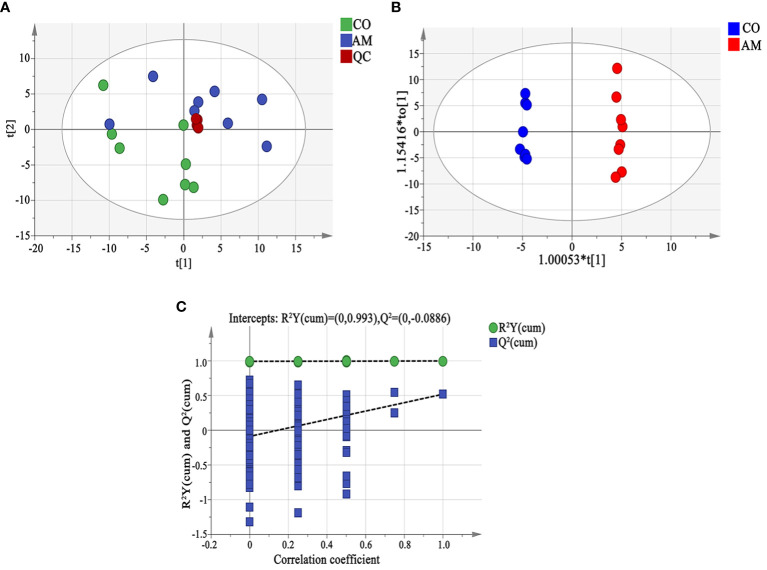
Fecal metabolomic analysis. **(A)** Principal component analysis (PCA). The distance of each coordinate point represented the degree of aggregation and dispersion between samples. A close distance indicated high similarity between the samples. PC1 and PC2 represented the contribution values of the first and second principal components, respectively. **(B)** OPLS-DA analysis displayed the grouped discrimination of the control and AM groups by the first two PCs. **(C)** OPLS-DA model validation. The abscissa representsed the replacement reservation degree of the replacement test. The ordinate represented the values of *R^2^* (green dot) and *Q^2^* (blue square), and the two dashed lines represented the regression lines of *R^2^* and *Q^2^*. QC, quality control.

The results of PCA analysis showed that the degree of dispersion in the AM group was greater than that in the control group. The cluster of QC samples in the PCA score plot demonstrated satisfactory stability and repeatability of the metabolic profiling method (**Figure 5A**). The OPLS-DA analysis found significant differences in the metabolites between the two groups (**Figure 5B**), and the predictive ability of the OPLS-DA model was verified reliable and no overfitting (*R^2^X*=0.458, Q^2^=0.518) (**Figure 5C**).

**Figure 6:**
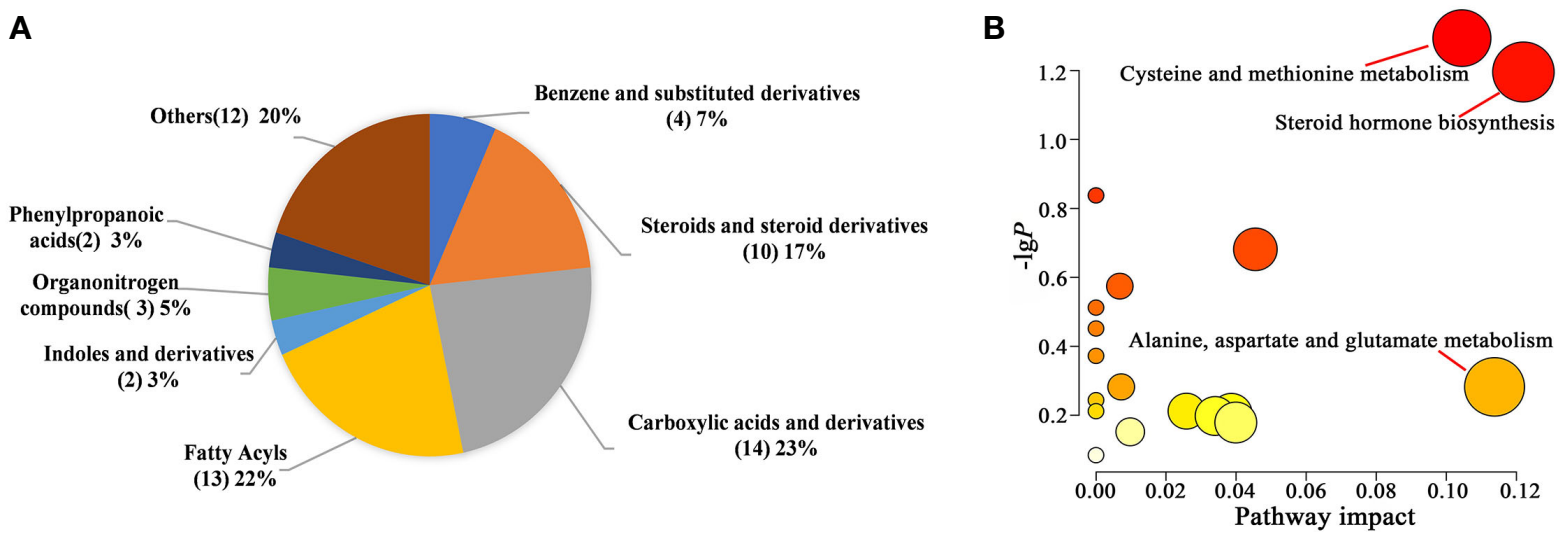
Differential metabolite analysis between the two groups. **(A)** Pie chart of HMDB subclass compounds. The different colors in each pie represented different HMDB classifications, and the area represented the relative proportion of metabolites in the classification. The number in the brackets represented the amount of corresponding metabolite class. **(B)** Pathway analysis of significantly altered metabolites.

We identified 60 metabolites that were differentially expressed between the control and AM groups *via* OPLS-DA analysis and t-test (VIP > 1.0, *P*<0.05) (**Supplementary Table S2**). Compared to the information in the Human Metabolome Database (HMDB, https://hmdb.ca/metabolites), the four types with the highest abundance are carboxylic acids and their derivatives carboxylic acids and derivatives (23%), fatty acyls (22%), steroids and steroid derivatives (17%), benzene and substituted derivatives. (7%) (**Figure 6A**). 36 metabolites were up-regulated in the AM group in comparison to the control group, while the rest metabolites represented the trend of down-regulation (**Supplementary Table S2**). The clustering heatmap of the differential metabolites was shown in **Supplementary Figure S1**. To systematically evaluate the perturbed metabolism in AM, the pathway analyses were performed. As a result, we found there were striking differences in steroid hormone biosynthesis, cysteine and methionine metabolism, and alanine, aspartate, and glutamate metabolism between the control and AM groups (**Supplementary Table S3**, **Figure 6B**).

### 3.6 Correlation between differential metabolites and gut microbiota in mice with adenomyosis

**Figure 7:**
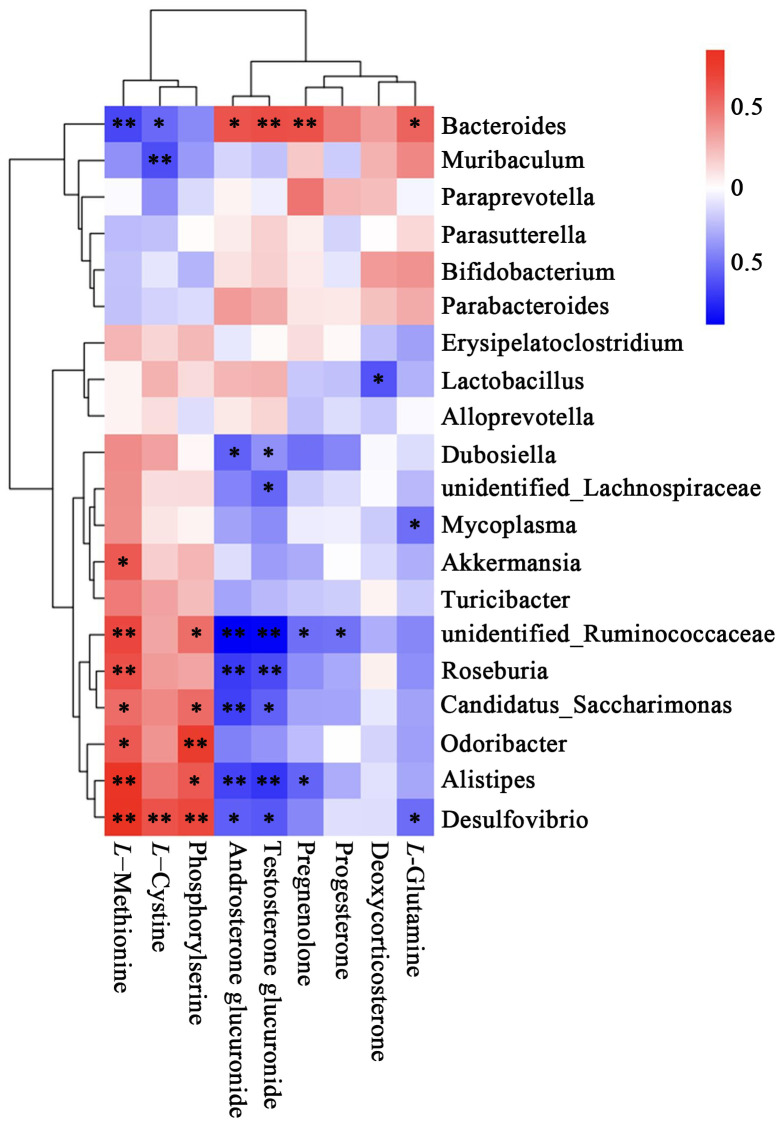
Heatmap of correlation between metabolites and top 20 relative abundance species at the genus level. The correlation (R) value is displayed in different colors in the figure. The legend on the right shows the range of colors for different R values, with red representing positive correlations and blue representing negative correlations. *P<0.05, **P < 0.01.

The results of the correlation analysis was shown in **Figure 7**. The top 20 species at the genus level and the 9 differential metabolites were included for analysis. The metabolites increased in the AM group, including *L*-methionine and *L*-cystine, were significantly negatively correlated with *Bacteroides* and positively correlated with *Desulfovibrio*. The metabolites decreased in the AM group, including Pregnenolone, Androsterone glucuronide, and Testosterone glucuronide, were negatively correlated with *unidentified_Ruminococcaceae* and *Alistipes*, whereas positively correlated with *Bacteroides*.

## 4 Discussion

As a complex and important part of the human body, the gut microbiota plays a role in the immune regulation and pathogens resistance of their host (Jandhyala et al., 2015; Sidhu and van der Poorten, 2017). One of the major ways the gut microbiota interacts with the host is through intestinal metabolites, small molecules produced as intermediate or end products of microbial metabolism (Zhang et al., 2022). The combined analysis of gut microbiota and intestinal metabolomics helps to understand the interaction between the intestinal flora and the host (Zhou et al., 2020). In this study, 16S rRNA sequencing and non-targeted metabonomic analysis methods were adopted to reveal the variation of the intestinal metabolomics and gut microbiota in mice with AM, and the relationship between them was further investigated by correlation analysis.

In terms of gut microbiota, we found that despite the similarity in alpha diversity and beta diversity, bacterial relative abundance varied between the two groups at phylum and genus levels. *Bacteroidetes* are the major members of the gut microbiome in AM mice, followed by *Firmicutes*, considered as the two most dominant bacteria in the gut microbiome at the phylum level (Yang et al., 2022). The appropriate ratio of the *Firmicutes*/*Bacteroidetes* has been supposed to benefit the homeostasis of the host, while the disturbance of the ratio may link to complications such as diabetes and inflammatory bowel disease (Stojanov et al., 2020; Yanez et al., 2021). *Firmicutes*/*Bacteroidetes* ratio is elevated in type 2 diabetes patients, and *Firmicutes* were positively correlated with proinflammatory gene expressions (Bahar-Tokman et al., 2022). Studies have also shown that the ratio of the two intestinal flora may change the body weight. Researchers demonstrated that obese mice have lower levels of *Bacteroidetes* and a higher proportion of *Firmicutes* than lean mice (Yang et al., 2017b). It has been proved that the proportion of *Firmicutes* to *Bacteroidetes* increased in both the genital and intestinal tracts of endometriosis (Shan et al., 2021), and similar trends were verified in our research. Besides, our study on AM model mice found that the weight gain of AM model mice was significantly higher than control mice, companies with the increased ratio of *Firmicutes*/*Bacteroidetes*. It is reported that the risk of clinically suspected endometriosis was higher among women who were overweight compared to normal weight (Rowlands et al., 2022), and the positive associations between endometriosis and body size in adulthood are evident (Rossi et al., 2021). Researchers believe there is pathophysiological interaction between endometriosis and obesity, especially in angiogenesis and inflammation (Pantelis et al., 2021). This may explain the significant increase in body weight of AM mice. We speculate that controlling metabolism through gut microbiota may be a potential therapeutic target for AM in the future.

In addition, *Lactobacillus*, a species of *Firmicutes*, was found upregulated at the genus in the AM group. The increased level of the *Lactobacillus* has been demonstrated to associate with disorder of the sex hormone, resulting in the high CA125 levels, severe pain, and infertility in endometriosis (Chang et al., 2022). The increased estrogen and low progesterone levels have been recognized to be responsible for the endometriotic disease (Seifert, 2020). In the liver, the estrogen binded with glucuronic acid or sulfate *via* UDP-glucuronyltransferase and sulfotransferase is eventually discharged from bile into the intestine. However, binding estrogens may be uncoupled by intestinal bacteria possessed β-glucuronase and β-glucosidase, reabsorbed into the bloodstream, from the intestine, forming the enterohepatic circulation of estrogens. Increased enterohepatic circulation can lead to the excess of estrogen, which is closely related to the occurrence and development of ectopic endometrium (Tian, 2022). *Lactobacilli* have been figured out to contain genes encoding β-glucuronase, which might explain why increased *Lactobacilli* contribute to endometriosis (Cao et al., 2020). We also found that *Lactobacillus* were significantly increased in AM group compared with the control group, and supposed that the gut microbiota may contribute to the imbalance of estrogen/progesterone in AM.

Although serum differential metabolites, including 3-hydroxybutyrate, glutamic acid, proline, and choline, have been confirmed in AM patients (Bourdon et al., 2021), the metabolic changes in the gut of AM have not been thoroughly studied. Our research focused on the changes of metabolites in the intestine, and the results revealed that the metabolic profiles of the AM group were significantly different from those of the control group.

Firstly, it was found in our research that the progesterone in AM was significantly reduced, along with the decrease of intermediate products including progesterone and prognenolone. Estrogen is a key promoter for endometriotic lesion growth and progression, whereas progesterone is a master regulator tightly controlling estrogen actions. The researchers claimed that the established inflammatory environment of endometriosis disrupted the balance of hormonal regulation and reduced coordinated progesterone responses (MacLean and Hayashi, 2022). Oral contraceptives containing progesterone have been tried as a treatment for endometriosis and proved effective in two-thirds of patients (Donnez and Dolmans, 2021). For example, Norethindrone acetate, typical progesterone, has been widely used in the treatment of pelvic pain and irregular bleeding in endometriosis by inhibiting ovulation and reducing the level of prostaglandinn (MacLean and Hayashi, 2022). It is also confirmed that pregnenolone sulfate, the intermediate product of progesterone, has been associated with a reduced risk of post-surgical pelvic pain in young patients with endometriosis (Sasamoto et al., 2022). So, we supposed that the decreased progesterone in the intestinal metabolism may be responsible for the access of AM.

In addition, a hypoandrogen state was found in AM mice, reflecting in the reduction of androsterone glucosidate and androsterone glucosidate, which are two major testosterone metabolites. There is growing evidence that androgens are key regulators of body fat distribution in both men and women (Tchernof et al., 2018). Studies have confirmed that BMI at the age of 18 is negatively correlated with androgens (dehydroepiandrosterone, dehydroepiandrosterone sulfate, androstenedione, testosterone) and 5α -glucuronic acid metabolites (Oh et al., 2021), the mechanism of which may attribute to the highly positive correlation between insulin resistance and low testosterone (Bianchi and Locatelli, 2018). Our study found that the down-regulated androsterone glucosidate was significantly positively correlated with Bacteroidetes. Therefore, we speculated that the weight gain of AM mice caused by the imbalance of the *Firmicutes*/*Bacteroidetes* ratio may be partly related to the down-regulation of androgen levels.

Besides, the upregulation of sulfur-containing amino acids, including phosphoserine, methionine, and cystine, was observed in AM group. Phosphoserine is a crucial intermediary in serine production (Mattaini et al., 2016). Serine can acquire sulfur delivered by methionine catabolism to form cysteine, which exists mainly in cystine (Cys-S-S-Cys) outside the cell. After being transported to the cell by cysteine transporters, cysteine participates in the synthesis of glutathione (GSH) due to its highly reduced state. As an antioxidant, GSH maintains cellular redox homeostasis, which is crucial in the development of tumors (Guo et al., 2021). Appropriately increased redox levels can support survival and proliferation by activating signaling pathways that can contribute to tumor growth. Therefore cancer cells need to maintain an intricate balance of antioxidant levels to survive, which helps to explain the increased biosynthesis of GSH in tumor cells and its positive correlation with high metastasis (Bansal and Simon, 2018). AM is characterized by a tumor-like malignant proliferation. Although there are few studies on the metabolic changes of GSH in AM, some researchers confirmed that in the myometrium of AM, glutamate glutathione and oxidized glutathione were increased, (Zhang et al., 2017; Song et al., 2022). Consistently, our research showed that methionine, cystine, and phosphoserine, significantly increased in the intestinal metabolites of AM mice, highlighting that increased synthesis of sulfur-containing amino acids may be a potential metabolic marker for abnormal AM proliferation.

Sulfate-reducing bacteria (SRB) are the main driving force of the sulfur biological cycle. *Firmicute Lactobacillus*, a kind of SRBS, has been upregulated in the AM mice in contrast to the control group. Researchers have demonstrated a positive correlation between cysteine concentration and *Firmicute Lactobacillus* abundance in the reproductive tract (Bloom et al., 2022). It may relate to the cysteine *β* -synthase and cysteine *γ* -lyase contained in the *Lactobacillus*, which contribute to the synthesis of cysteine from serine and methionine by a reverse super sulfur pathway (Matoba et al., 2020). Meanwhile, *Enterobacteriaceae*, another kind of SRB, was found to be upregulated in AM mice. Studies have verified that the *Enterobacteriaceae* can activate sulfate transport by sulfate osmosis enzyme and participate in sulfite reduction reaction *via* NADPH-sulfite reductase under aerobic conditions, contributing to the production of cysteine (Wang, 2022). Increased *Desulfovibrio* of SRB has also been presented. *Desulfomycin* contained in *Desulfovibrio*, reducing sulfate to H_2_S, has been demonstrated to be a crucial sulfite reductase in anaerobic conditions (Kushkevych et al., 2020). At the same time, sulfide may have a reverse-regulating effect on the flora. It was found that adding sulfide elevates the abundance of *Firmicutes* and *Desulphurvibrio* (Zhao et al., 2020),while cysteine uptake inhibition selectively reduces the growth of *Lactobacillus in vitro* (Bloom et al., 2022). Consistent with the existing studies, we found that *Enterobacteriaceae* and *Desulfurvibrio* were positively correlated with up-regulated sulfur-containing amino acids in AM, suggesting the potential relations between the SRB and increased metabolism of sulfur-containing amino acids. However, its specific mechanism needs further verified.

In conclusion, this study performed a comprehensive analysis of gut microbes and metabolites in AM mice by 16S rRNA sequencing and fecal metabolomics. AM mice have unique gut microbiota and metabolites, and the alteration in gut microbes may contribute to the regulation in metabolites. It provides different insights into the pathogenesis of AM from new approaches and perspectives.

The present study has some limitations. First, the relatively small sample size might lead to low statistical power. Second, there are certain restrictions on the research of the illness process because only a mouse model was employed. Thirdly, follow-up verification has yet to be conducted in this experiment. Further research will be done to confirm in the future.

## Data availability statement

The datasets presented in this study can be found in online repositories. The names of the repository/repositories and accession number(s) can be found below: NCBI, PRJNA895179.

## Ethics statement

The animal study was reviewed and approved by Ethical Committee in Research Medical College of China Three Gorges University of Medical Sciences.

## Author contributions

Conception and design: MZ and XF. Perform the experiments: SL, MC and YL. Performed the microbiome and metabolomic analysis: PC and KW. Drafted the manuscript: PC. Final approval of the completed manuscript: MZ and XF. All authors contributed to the article and approved the submitted version.
