# Supplementary material for: An insight into gut microbiota and metabolites in the mice with adenomyosis
Source: Front Cell Infect Microbiol. 2023 Feb 27;13:1075387. doi: 10.3389/fcimb.2023.1075387 (PMC10008959; doi:10.3389/fcimb.2023.1075387)

**Supplementary Figure S1** The clustering heatmap of the differential metabolites altered between AM group and control group. Red meant increased content, and blue means decreased content.


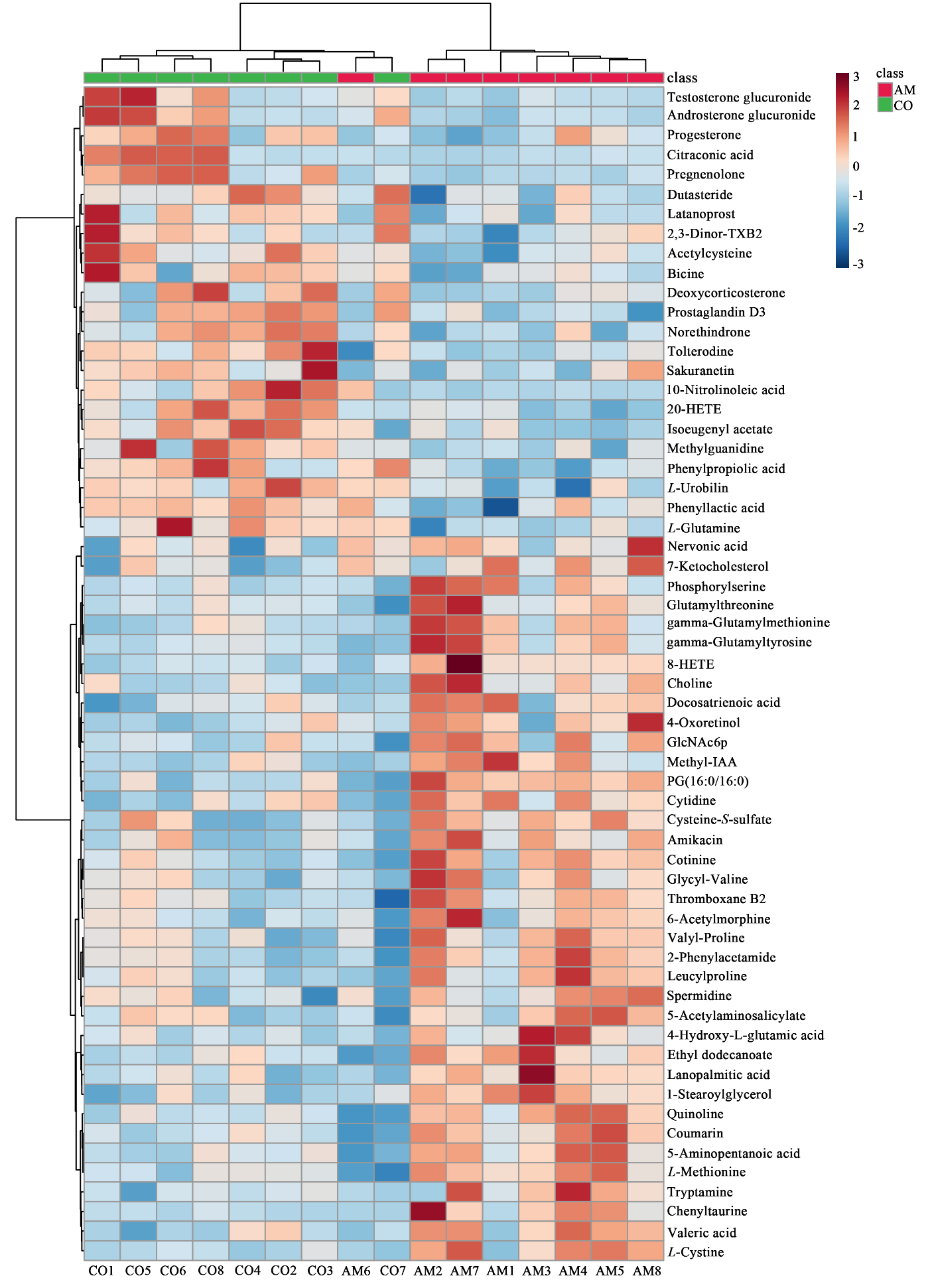

Supplement: Supplementary file 1 [file Table_1.docx]
